# Primary central nervous system lymphoma mimicking recurrent depressive disorder: A case report

**DOI:** 10.3892/ol.2015.2963

**Published:** 2015-02-13

**Authors:** WEIBO LIU, JING XUE, SHAOHUA YU, QIAOZHEN CHEN, XIUZHEN LI, RISHENG YU

**Affiliations:** 1 Department of Psychiatry, Second Affiliated Hospital of Zhejiang University School of Medicine, Hangzhou, Zhejiang 310009, P.R. China; 2 Department of Rheumatology, Second Affiliated Hospital of Zhejiang University School of Medicine, Hangzhou, Zhejiang 310009, P.R. China; 3 Department of Pathology, Second Affiliated Hospital of Zhejiang University School of Medicine, Hangzhou, Zhejiang 310009, P.R. China; 4 Department of Radiology, Second Affiliated Hospital of Zhejiang University School of Medicine, Hangzhou, Zhejiang 310009, P.R. China

**Keywords:** primary central nervous system lymphoma, magnetic resonance imaging, diffuse large B cell lymphoma, rheumatoid arthritis, depressive disorder

## Abstract

Primary central nervous system lymphoma (PCNSL) is a rare subtype of extranodal non-Hodgkin lymphoma, which is limited to the central nervous system. Few studies are available reporting psychiatric symptoms as the initial and dominant presentation of PCNSL. The current study reports the case of a PCNSL patient with a history of major depressive disorder and coexisting rheumatoid arthritis (treated with methotrexate), who initially presented with recurrent depressive disorder that showed no response to antidepressant drug therapy. Magnetic resonance imaging revealed multiple mass lesions in the brain, and pathological examination of the biopsy confirmed the diagnosis of diffuse large B cell lymphoma of the central nervous system. The present case demonstrated that PCNSL may affect mood in the early stages of the disease and thus, clinicians must be aware of this manifestation in patients with depressive disorder co-existing with immunosuppressive conditions, as early detection and appropriate treatment are important prognostic factors for PCNSL.

## Introduction

Primary central nervous system lymphoma (PCNSL) is a rare hematopoetic tumor, which accounts for <1% of all brain tumors, that arises within and is limited to the central nervous system (1). The incidence rate of PCNSL is <5 cases per 1 million people, this incidence appears to be increasing (2,3) An important risk factor for PCNSL is an alteration of the function of the immune system, and HIV infection is a powerful risk factor for acquiring PCNSL (2). The outcome of PCNSL remains unsatisfactory with a survival of <20–30% after 5 years and a median survival of 10–20 months (3). The disease is highly malignant and may rapidly lead to mortality if diagnosis and treatment are delayed (1,4). The clinical manifestation of PCNSL is variable; patients typically exhibit cognitive decline and headaches (5,6) and may present with psychiatric symptoms in combination with focal neurological deficits, including aphasia, hemiparesis and ataxia (7). Although the majority of patients respond to radiotherapy, relapse is almost inevitable (4). Chemotherapy is applied for the treatment of relapse after radiotherapy, however, in elderly patients a high risk of treatment-related complications exists (6).

The present study reports the case of a 58-year-old patient with a history of major depressive disorder (MDD) and coexisting rheumatoid arthritis, who presented with recurrent depressive disorder, fatigue, dizziness, vomiting and a staggering gait. Written informed consent was obtained from the patient.

## Case report

In March 2012, a 58-year-old male was referred to the inpatient psychiatric service of the Second Affiliated Hospital of Zhejiang University School of Medicine (Hangzhou, China) with depressive symptoms. Six years previously, January 2006, the patient had been diagnosed with major depressive disorder (MDD), which had improved through treatment with fluoxetine. Subsequently, MDD recurred twice (August 2007 and February 2009), each ocassion lasting for two months, and a favorable response to fluoxetine (20 mg/day) was exhibited. The patient had ceased fluoxetine treatment voluntarily two years prior to the current admission (January 2010), due to full recovery from depression. However, four months prior to this admission (December 2011), further fluoxetine treatment (20 mg/day) was prescribed by a local psychiatric clinic after the patient presented with depressed mood and anhedonia. One month later, the patient complained of unimproved depression, fatigue and occasional vomiting, and was prescribed an increased dosage of fluoxetine (40 mg/day); however, the patient also experienced dizziness concurrent with the depressive symptoms. In addition, the patient been diagnosed with rheumatoid arthritis, for which methotrexate (10 mg once a week) and celecoxib (200 mg/day) were administered intermittently for two years; the patient’s symptoms fluctuated during this time.

The neurological examination showed staggering gait with normal motor strength of the extremities. No cerebellar ataxia, peripheral neuropathy or positive Babinski reflexes were observed. A mental status examination revealed depressed mood with suicidal ideation, psychomotor retardation and lack of energy. The patient exhibited fatigue and mental dullness during the examination. Psychological testing indicated severe depression, with a score of 29 on the 17-item Hamilton Rating Scale for Depression (8), and cognitive impairment, with a Mini Mental State Examination (9) score of 20/30.

**Figure 1:**
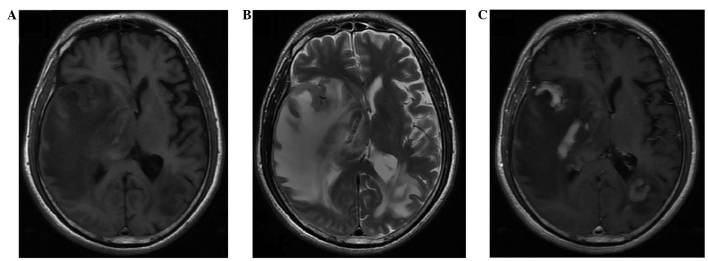
Brain MRI: Axial MRI revealed multiple expansive lesions in the right temporoparietal lobe and left parietal-occipital lobe, which showed (A) hypointensity on axial T1-weighted images, (B) hyperintensity on axial T2-weighted images and (C) homogeneous enhancement on T1-weighted contrast-enhanced MRI. The majority of the lesions showed C-shape enhancement on T1-weighed contrast-enhanced MRI. Extensive perifocal edema was also observed. MRI, magnetic resonance imaging.

The initial diagnosis was recurrent depressive disorder, however, a thorough physical examination was conducted to exclude the possibility of somatic or neurological origin. Blood analysis including hemocytology, liver and kidney function, antinuclear antibodies and thyroid hormone were all within the normal ranges. However, rheumatoid factor (66.8 IU/ml; normal range, 0–25 IU/ml), C-reactive protein (30.8 mg/l; normal range, 0–10 mg/l) and cyclic citrullinated peptide (800 IU/ml; normal range, 0–25 IU/ml) were shown to be elevated. The serum toxicology screen, human immunodeficiency and serum syphilis antibody tests were negative. Magnetic resonance imaging (MRI) of the brain revealed multiple lesions with marked edema in the temporal, parietal and occipital lobes (Fig. 1).

**Figure 2:**
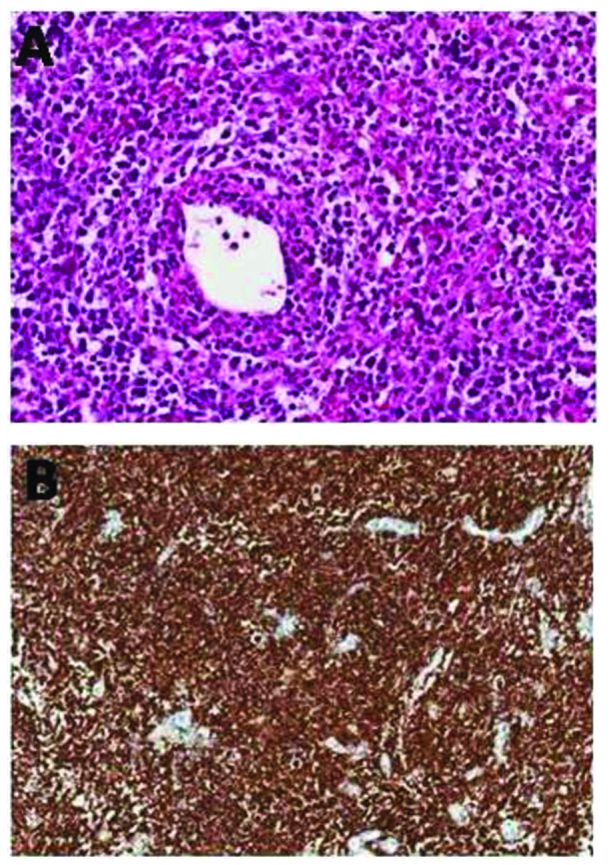
Immunohistochemical staining of the brain-biopsy specimen. (A) Diffuse atypical large lymphocytic tumor cells are visible, with penetration into and through the vessel wall (H&E staining, magnification ×200). (B) CD20 staining reveals diffuse and strong cytoplamic staining (magnification, ×100).

These findings indicated the presence of brain tumors, and the patient was transferred to the inpatient neurosurgical service. Fluoxetine treatment was discontinued, and a craniotomy was conducted for open biopsy and resection of the temporal lobe lesion. A pathological examination of the biopsy specimen confirmed the diagnosis of diffuse large B cell lymphoma, and immunohistochemical staining revealed that the tumor cells were positive for CD20 and bcl-6 and negative for CD3 and Epstein-Barr virus (EBV)-EBV-encoded non-polyadenylated RNA (Fig. 2). A positron emission tomography scan was negative for systemic lymphoma, and a slit lamp examination revealed no evidence of ocular lymphoma. Therefore, a final diagnosis of PCNSL was determined. The patient received whole brain radiotherapy (45 Gy total, administered in 21 fractions, five times a week) following surgery. During the one year follow-up, no clinical or radiographic evidence of PCNSL recurrence was observed, and the patient’s mental state was stable without antidepressant therapy. No recurrence or worsening of MDD has been observed to date.

## Discussion

PCNSL is a highly malignant disease which may rapidly lead to mortality if diagnosis and treatment are not immediately administered (1,4). A variety of clinical manifestations may be observed in PCNSL patients, including psychiatric symptoms in combination with focal neurological deficits (7,10,11). One study found that 43% of patients presented with neuropsychiatric symptoms during the course of PCNSL (7). However, a number of studies have reported psychiatric symptoms as the initial and dominating presentation in PCNSL. Melinz *et al* (12) reported a PCNSL patient presenting with mania, whilst Fisher *et al* (13) reported PCNSL involving the limbic system, presenting with depression and intermittent vomiting.

The present study describes a case of PCNSL mimicking recurrent depressive disorder in a 58-year-old male with a past history of MDD; the psychiatric symptoms of PCNSL were difficult to distinguish from common psychiatric disorder, and therefore delayed the diagnosis. However, other symptoms, including fatigue, dizziness, vomiting and a staggering gait indicated that the initial diagnosis of MDD recurrence may be incorrect. These symptoms, concurrent with the lack of response to antidepressant drugs that had previously been an effective treatment in this patient, suggested the possibility of another medical condition.

As with all masses in the CNS, the location of PCNSL lesions determines the clinical presentation (7). The neuropsychological changes appear to be associated with diffuse involvement of the periventricular white matter or the corpus callosum by a tumor (14). In the current study, MRI revealed that the tumor had invaded a number of brain regions, primarily located in the temporal, parietal and occipital lobes. As the temporal lobe is a part of the limbic system, which is involved in regulating emotion (15), it is possible that the infiltration of the tumor into the temporal lobe may have caused the recurrent depressive symptoms in this case.

An important risk factor for PCNSL is an alteration of the function of the immune system; however, PCNSL may occur in immunocompetent or immunocompromised patients (16,17). Long-term immunosuppressive therapy or an underlying disease with associated immunosuppression are high-level risk factors for PCNSL (10). In the present study, the patient suffered from active rheumatoid arthritis, for which he was treated with an immunosuppressant over a period of two years. Drug- or disease-induced immunosuppression may increase the risk of developing PCNSL. Therefore, the possibility of PCNSL must be considered when assessing patients with depressive symptoms who have coexisting immunosuppressive conditions or are receiving immunosuppressive therapies. Vigilance is essential during diagnostic evaluation and a thorough examination for signs of physical disorder must be conducted. In particular, MRI of the brain is useful for the detection of PCNSL presenting with initial psychiatric symptoms.

In conclusion, the present case demonstrates that PCNSL may affect mood in the early stages, and patients may present with depression as the initial and dominating symptom. Brain MRI is a useful adjuvant examination for the detection of depression that is caused by other physical disorders, and must be used in patients with the clinical manifestation of MDD who have coexisting immunosuppressive conditions, to allow for the early detection of PCNSL and an improved prognosis.
